# Tumor microenvironment and fibroblast activation protein inhibitor (FAPI) PET: developments toward brain imaging

**DOI:** 10.3389/fnume.2023.1183471

**Published:** 2023-07-18

**Authors:** Mehdi Djekidel, Rahaf Alsadi, Maya Abi Akl, Othmane Bouhali, Jim O’Doherty

**Affiliations:** ^1^ Department of Radiology/Nuclear Medicine, Northwell Health, New York, NY, United States; ^2^ Division of Arts and Science, Texas A&M University at Qatar, Doha, Qatar; ^3^ Department of Electronics and Information Systems, Medical Image and Signal Processing (MEDISIP), Ghent University, Ghent, Belgium; ^4^ Qatar Computing Research Institute, Hamad Bin Khalifa University, Doha, Qatar; ^5^ Siemens Medical Solutions, Malvern, PA, United States; ^6^ Department of Radiology & Radiological Sciences, Medical University of South Carolina, Charleston, SC, United States; ^7^ Radiography and Diagnostic Imaging, University College Dublin, Dublin, Ireland

**Keywords:** PET, FAPI imaging, neuro-oncology, nuclear medicine, radiotracer

## Abstract

Fibroblast activation protein (FAP) is a type-II membrane bound glycoprotein specifically expressed by activated fibroblasts almost exclusively in pathological conditions including arthritis, fibrosis and cancer. FAP is overexpressed in cancer-associated fibroblasts (CAFs) located in tumor stroma, and is known to be involved in a variety of tumor-promoting activities such as angiogenesis, proliferation, resistance to chemotherapy, extracellular matrix remodeling and immunosuppression. In most cancer types, higher FAP expression is associated with worse clinical outcomes, leading to the hypothesis that FAP activity is involved in cancer development, cancer cell migration, and cancer spread. Recently, various high selectivity FAP inhibitors (FAPIs) have been developed and subsequently used for positron emission tomography (PET) imaging of different pathologies. Considering the paucity of widely available and especially mainstream reliable radioligands in brain cancer PET imaging, and the poor survival rates of patients with certain types of brain cancer such as glioblastoma, FAPI-PET represents a major development in enabling the detection of small primary or metastatic lesions in the brain due to its biological characteristics and low background accumulation. In this work, we aim to summarize the potential avenues for use of FAPI-PET, from the basic biological processes to oncologic imaging and with a main focus on brain imaging.

## 1. Introduction

Brain tumors are frequently associated with poor outcomes, with a 5-year relative survival for all malignant brain tumors of around 36%, and 7% specifically for glioblastoma (1). Imaging plays a major role in guiding treatment and is indispensable in medical decision making. Magnetic resonance imaging (MRI) performs relatively well, however presents limitations in evaluation of tumor boundaries and post-treatment changes which can be challenging to estimate accurately (2). There are also limits in determining viable tumor from surrounding inflammatory and non-inflammatory changes (i.e., due to post treatment effects from therapeutic interventions: i.e., surgery, chemotherapy or radiation) (3). Contrast-enhanced MRI is mostly a function of blood-brain-barrier (BBB) integrity and tumor vascularity and thus is a non-specific form of tumor mass characterization prone to equivocal interpretation. Accurate assessment of various tumor characteristics and parameters is fundamental to improve management and outcomes.

Positron emission tomography (PET) has been used in the evaluation of brain tumors for many years (4), showing utility in several instances such as improving delineation of tumor boundaries for surgery and radiotherapy planning (5), differentiating between tumor progression and treatment-related changes (6), monitoring early/late therapy response (7), and identification of favorable biopsy sites (8). Historically, evaluation of glucose metabolic rate (^18^F-FDG) has been used in assessing brain tumors, with sensitivity being hindered by elevated normal background uptake of ^18^F-FDG, as well as increased glucose usage due to inflammatory responses potentially independent to the tumor response or growth.

As PET evolves, ^18^F-FDG is slowly becoming supplanted by amino-acid imaging (when available), with radioligands such as ^11^C-MET, ^18^F*-*FET, and ^18^F-DOPA (9). Along with the crucial advantage that amino acid tracers can easily permeate across the BBB (whether the BBB is compromised or not), this pathway is thus more specific in highlighting “live” proliferating cells rather than structural changes. On the other hand, FAPI ligands do not cross the BBB and hence may offer an additional complementary representation of tumor characteristics (10, 11). Other amino acid radioligands such as α-[^11^C]-methyl-l-tryptophan (AMT) and ^18^F-Fluciclovine (FACBC), as well as glutamine-based amino acids are under evaluation for their potential in tumor characterization, prognostication, viable volume delineation and detection of recurrence in brain tumors and metastases (12).

Other uptake pathways have been explored such as the thymidine nucleoside analogue using i.e., (^18^F-FLT), which reflects cellular proliferation (13). ^18^F-FLT is dependent on disruption of the BBB for successful tracer uptake, and thus is a limitation compared to amino acid-based tracers. Hypoxia imaging ligands (^18^F-FMISO, ^18^F-FAZA, ^18^F-HX4, ^64^Cu-ATSM) have also displayed limitations such as a low target-to-background ratio, poor clearance times, and none have made a successful translation to routine clinical practice (14). Considering neuroinflammation has been explored as a hallmark of several neurological diseases, it has also been postulated to be an important element of neuro-oncology assessments. Neuroinflammation investigations has included identification of elevated expression of translocator protein (TSPO) in glioblastoma (GBM), a primary brain tumor with a high level of aggressiveness, in part due to neuroinflammation and microglial activation hinting at a pivotal role of TSPO in tumorigenesis and progression (15). Radiotracers such as ^11^C-PK11195, ^18^F-DPA-714 and ^18^F-GE-180 show promising results (16–18), but lack large scale clinical trials to prove efficacy. Other uptake routes such as poly(ADP ribose) polymerase (PARP) enzymes which are critical for DNA repair, are under development. In comparison to amino acid-based tracers, PARP tracers have demonstrated good uptake in brain tumors with a lower background uptake in the cortex (19), and preclinical investigations of 3 different PARP radioligands (^18^F-PARPi, ^18^F-FTT and ^18^F-FPyPARP) investigated imaging pharmacokinetics (20), however still require small molecules to cross the BBB. Chemokine receptor-4 (CXCR4) have been shown to exhibit affinity for glioma cells, and tracers such as ^68^Ga-Pentixafor have demonstrated higher tumor uptake compared to amino acid tracers such as ^18^F-FET (21). Use of somatostatin receptor (SSTR) targeting for treating brain tumors has also been investigated (using ^90^Y-DOTATOC) (22), although remains investigational. Studies have also confirmed prostate specific membrane antigen (PSMA) expression in the tumoral vessels of high-grade gliomas (23). With the advent of low background uptake in the normal brain tissue and a high tumor-to-brain ratio of ^68^Ga-PSMA and a theranostic pair of ^177^Lu-PSMA, recent efforts have been made to exploit this mechanism for treatment, showing good imaging characteristics but a limited use of ^177^Lu-PSMA (24).

In lieu of the aforementioned, imaging the tumor microenvironment can be an essential tool in the evaluation of these patients as it gives a complete picture of the patient's disease status at diagnosis and throughout the patient care continuum. This has proven to be extremely useful with novel ligands such as fibroblast activated protein inhibitors (FAPI) PET (25).

FAPI-PET has been shown to be very useful in many non-oncological indications (26), including:

• Cardiovascular diseases (27–29): FAPI PET has been assessed in patients with myocardial infarction, myocardial fibrosis and metabolic syndrome (27, 30–33).
• Immune mediated diseases such as Crohn's disease or Rheumatoid Arthritis (34–36)
• FAPI PET is undoubtedly a promising future PET radiopharmaceutical for solid tumors in various cancers (37–47).

Taking into consideration the great potential of FAPI-PET and taking note of the fact that the tumor microenvironment has also been evaluated with FAPI-PET in brain tumors (48–50). We propose to review the benefits, limitations, and potential indications of FAPI PET in brain tumors.

## 2. Background: fibroblasts and imaging

### 2.1. The tumor microenvironment

Many tumor types are characterized by a strong desmoplastic reaction, i.e., they are subject to causing or forming adhesions or fibrous connective tissue within a tumor. Tumors can be considered to comprise two parts, the malignant cells and the stroma (containing components such as basement membrane, fibroblasts, extracellular matrix, immune cells, and vasculature), also known collectively as the tumor microenvironment. While tumor microenvironment may vary with different tumor types, a hallmark feature includes a tumor mass with up to 90% of cancer associated fibroblasts (CAFs—a cell that synthesizes extracellular matrix & collagen and produces the structural tissue framework) and extracellular fibrosis, leaving the original tumor cells in the minority (51–53).

### 2.2. Fibroblast activation protein-α (FAP) characteristics

Fibroblast activation protein-ɑ (FAP) is a type II transmembrane glycoprotein, specifically expressed on the surface of activated fibroblasts. It is a 760 amino acid serine protease, where the active site is localized in the extracellular part of the protein, and is catalytically active as a 170 kD homodimer. Normal fibroblasts have shown low FAP expression in normal human tissues, however in activated fibroblasts, expression of FAP is increased in the presence of tissue damage, remodeling, or inflammation (54). Furthermore, FAP expression is seen on activated CAFs of more than 90% of epithelial carcinomas (54). Through crosstalk with surrounding cells of the tumor microenvironment, CAFs play an essential role in tumor proliferation, metastasis, neoangiogenesis, extracellular matrix remodeling and immunosuppression (37).

### 2.3. Molecular imaging of FAP

Differential expression of FAP in normal tissue compared with tumors or inflammation allows FAP to become a candidate for molecular imaging, which can be performed by linking FAP inhibitors (FAPIs), peptides or antibodies to radionuces using chelators for use in PET/CT, PET/MR, SPECT/CT or SPECT/MR imaging. Through the development of FAPIs, the exploration of FAPI-PET imaging and FAPI-based radioligand therapy for different tumor types and some non-oncological diseases is gaining momentum (38, 39). Various derivatives of FAPI have been developed with the aim to improve affinity for FAP and demonstrate better pharmacokinetic properties. Examples include FAPI-02 (40), FAPI-04 (41), DATA5m.SA.FAPI (42), and DOTA-2P(FAPI)2 (43) which have demonstrated promising results in clinical studies, and other derivatives are at preclinical imaging stages, such as ^18^F-fluoroglycosylation-FAPI (44), QCP01, QCP02 (45), and AAZTA5.SA.FAPI (46). The most common radioisotopes used in clinical FAPI imaging development are ^18^F and ^68^Ga, with ^99m^Tc ligands also being recently developed for situations where PET imaging is not available. ^68^Ga labeled FAPIs provide the more favorable imaging features in terms of high detection rate in a variety of tumors, even in cases considered to be challenging for ^18^F-FDG PET (47).

## 3. FAPI PET in oncology

A recent review summarized many of the most common FAPIs used in clinical oncological research studies (55) and some examples of clinical studies are detailed in Table 1. Much research is focused on chemical modification of inhibitors to obtain derivatives with higher affinity for FAP, in turn improving imaging characteristics and pharmacokinetics, with a recent paper evaluating 11 derivatives in animal models (56).

**Table 1:** Summary of some FAP inhibitors used in a selection of clinical oncology research studies. It should be noted that FAPI-PET has so far been explored primarily in single-center studies, in heterogeneous and small cohorts of oncology patients.

Radioligand	No. of patients & tumor types	Primary finding(s)
68Ga-FAPI-21 & 68Ga-FAPI-46 & 68Ga-FAPI-04 (56)	Various cancers (3 pts 68Ga-FAPI-21, 3pts 68Ga-FAPI-46 & 4 pts 68Ga-FAPI-04)	FAPI-46 showed improved tumor-to-organ ratios, resulting in enhanced image contrast. Depending on the tumor type, tracer accumulation is prolonged by FAPI-46
^68^Ga-FAPI-04 (25)	Various cancers (80 pts)	Several highly prevalent cancers presented with remarkably high uptake and image contrast on ^68^Ga-FAPI PET/CT.
Al^18^F-NOTA-FAPI (57)	Various cancers (10 pts)	Lower radiation dose compared to ^68^Ga-FAPI-04, ^68^Ga-FAPI-46 and ^68^Ga-FAPI-74. No bone uptake, and detected more lesions than ^18^F-FDG in several patients
^68^Ga-DOTA.SA.FAPi (58)	Various cancers (54 pts)	High target-to-background ratio in various types of cancers. Clear advantages against ^18^F-FDG in brain metastases.
^68^Ga-DOTA-2P(FAPI)_2_ (43)	1 × nasopharangeal carcinoma, 1 × thyroid carcinoma, 1 × hepatocellular carcinoma (3 pts)	The tumor uptake of ^68^Ga-DOTA-2P(FAPI)2 was approximately 2-fold stronger than that of ^68^Ga-FAPI-46 in patient-derived xenografts
99mTc-FAPI-34 (59)	1 × pancreatic, 1 × ovarian (2 pts)	Useful tracer for diagnostic scintigraphy when PET is not available. Chelator easily adapted to 188Re or 90Y therapeutic applications
68Ga-FAPI-02 & 68Ga-FAPI-04 (49)	Glioma (2 pts FAPI-02, 16 pts FAPI-04)	Increased tracer uptake may allow distinction between low-grade IDH-mutant and high-grade gliomas.
68Ga-FAPI-74 & 18F-FAPI-74 (60)	Lung cancer (1 pt 68Ga-FAPI-04, 9pts 18F-FAPI-74)	High contrast and low radiation burden of FAPI-74 PET/CT favor multiple clinical applications. Results supported target volume definition for guiding radiotherapy
68Ga-FAPI-46 & 68Ga-FAPI-04 (61)	Pancreatic ductal carcinoma (PDAC, 19 pts)	68Ga-FAPI PET-CT led to restaging in half of the patients with PDAC and most patients with recurrent disease compared with standard of care imaging

It serves to summarize some of the results from Table 1. A retrospective analysis of 80 patients (28 different cancers) demonstrated high uptake of ^68^Ga-FAPI-04 with low background uptake and determined that tumor-to-background contrast ratios were between 3-fold and 6-fold higher depending on a low, medium and high uptake group (25). Related work showed that FAPI-PET in pancreatic carcinomas leads to significant changes in tumor staging compared to routine contrast enhanced CT (CECT) (61), with 7 of the 19 patient cohort having changes in treatment management (i.e., alteration of treatment type, treatment intent or changes within a treatment regime).

### 3.1. Radiation dosimetry and uptake time

Regarding radiation dosimetry and imaging times in FAPI-PET, a recent study of 10 lung cancer patients, longitudinal PET scans of ^18^F-FAPI-74 demonstrated an ideal imaging time-point of 1 h post-injection and a good radiation dosimetry profile (effective dose = 1.4 + −0.2 mSv) (60). A related biodistribution study showed good efficacy with ^18^F-NOTA-FAPI, demonstrating low physiological bone uptake, and was able to detect more lesions than ^18^F-FDG in several patients also with 1 h uptake (57). Another study directly compared ^18^F-FDG to ^68^Ga-DOTA.SA.FAPi imaging in 54 patients (14 types of cancers), also showing a good radiation dosimetry profile (effective dose = 0.011 mSv/MBq), and demonstrated a higher contrast to noise in comparison to the ^18^F-FDG images for certain cancers and a similar uptake time (58). Imaging of ^68^Ga-DOTA-2P(FAPI) in 3 patients also demonstrated an effective dose = 0.019 mSv/MBq. In preclinical studies, tumor uptake of ^68^Ga-DOTA-2P(FAPI) was approximately 2-fold higher than that of ^68^Ga-FAPI-46 in patient-derived xenografts. Results in patients demonstrated a significantly higher tumor uptake of ^68^Ga-DOTA-2P(FAPI)2 than of ^68^Ga-FAPI46 in all tumor lesions (SUV_max_, 8.1–39.0 vs. 1.7–24.0, respectively) at similar post-injection imaging times (57). Although research studies utilize a 1 h uptake time for the majority of FAPI-PET agents, serial timepoint imaging in brain FAPI-PET is still commonly performed in order to evaluate an ideal imaging timepoint (63).

## 4. FAPI-PET in neuro-oncology

### 4.1. Rationale

Although brain tumor PET can also be performed using radioligands exploring a host of other uptake routes such as amino acids, somatostatin receptors, TSPO, CXCR4, PSMA and hypoxia, so far there are no specific clinical studies involving a direct comparison of FAPI-PET to non-FDG radioligands in the brain. A range of studies have shown a significantly lower FAPI background distribution in the brain relative to other radiotracers evaluating brain tumors, especially ^18^F-FDG (62–64). This makes FAPI a promising radiotracer in the detection and evaluation of tumors and lesions naturally exhibiting low to moderate uptake of ^18^F-FDG including primary and secondary/metastatic brain lesions as well as post-treatment changes. Favorable early clinical trial results demonstrate the potential for FAPI to outperform or even complement other radiotracers.

### 4.2. Protocol

In FAPI-PET for neuro-oncology applications, the protocol consists of various aspects: patient preparation, radiopharmaceutical preparation, performing imaging and subsequent image analysis & reporting findings.

a. No specific patient preparation is required other than adequate hydration and voiding in order to decrease the radiation exposure to the patient.
b. FAPI radiopharmaceutical is radio-synthesized, whereby FAPI is coupled to a radionuclide of choice (most commonly ^68^Ga or ^18^F) via a chelator to produce the radiopharmaceutical. A range of experimental radiotracers exist, the use of which are institution dependent until regulatory approval of specific FAPI-PET agents (see Table 1). Labeling with ^18^F has also been performed, solving logistical issues with the short half-life of ^68^Ga, improving transport availability/distribution, automated radiochemistry, and also improving the image spatial resolution due to a shorter positron pathlength.
c. Patient is administered the radiopharmaceutical intravenously. Uptake phase varies from 15 to 60 min, with images frequently acquired around 30–60 min post-administration of the tracer. Highest contrast was achieved in primary tumors, lymph nodes, and distant metastases at 1 h after injection with many FAPI radioligands, but should be optimized to the specific scanner used and ideally chosen based on published data specifically to the radioligand of interest (50).
d. Literature demonstrates that administered activities for clinical cohorts are within 200–300 MBq for all ^18^F and ^68^Ga FAPI radioligands, both with favorable radiation dosimetry and good image quality. Image reconstruction varies widely depending on the PET scanner used, and routine clinical parameters for either ^18^F or ^68^Ga radioligands currently should be used as a first iteration.
e. Interpretative criteria include traditional qualitative visual assessments with positivity defined as uptake above background. Semi-quantitative methods using standardized uptake values (SUV) measurements and corrections compared to contralateral cerebral uptake have been described.

### 4.3. Diagnosis

**Figure 1:**
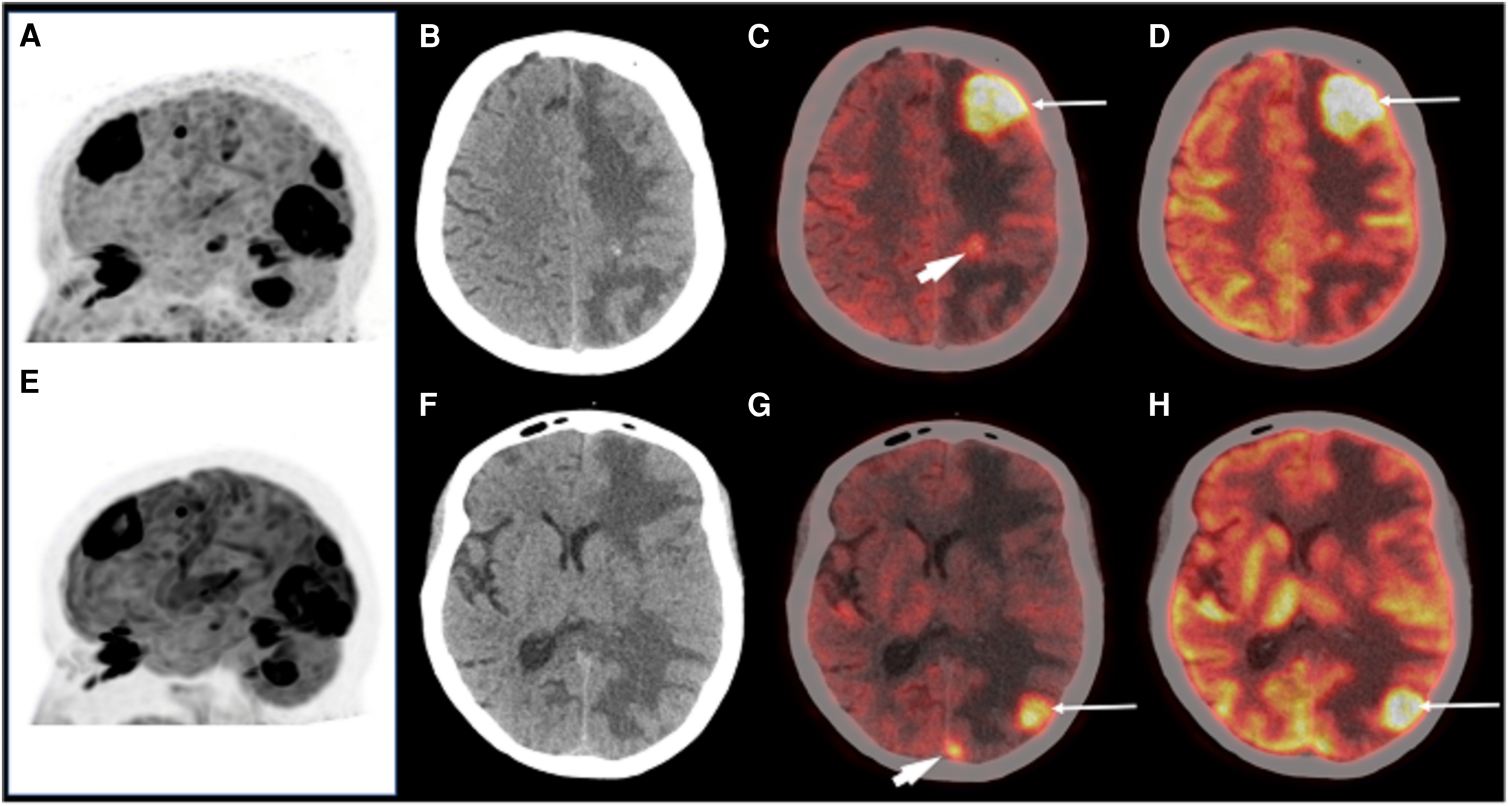
A patient with brain metastases imaged with ^68^Ga-DOTA.SA.FAPi (**A,C,G**) and ^18^F-FDG (**E,D,H**) for restaging. MIP images (**A,E**) showing multiple foci of intense tracer uptake in both. Axial CT revealed ill-defined isodense lesions in the left frontal, parietal, and occipital lobes with perilesional edema (**B,F**). Images showing increased ^18^F-FDG uptake in these lesions (**C,D,G,H** arrows). ^68^Ga-DOTA.SA.FAPi also revealed small occult lesions in the left parietal and left occipital lobe (**C,G** arrowhead). Reproduced with permission from (58).

In a recent first-in-man trial comparing ^18^F-FDG to ^68^Ga-DOTA.SA.FAPi imaging in a cohort of patients with secondary brain metastases, quantification of tracer uptake demonstrated higher image contrast with better SUL_peak_ (peak lean body mass corrected SUV) and SUL_avg_ (average lean body mass corrected SUV) and higher brain metastases-to normal brain parenchyma ratios observed on ^68^Ga-DOTA.SA.FAPi compared to ^18^F-FDG (SUL_peak_: FAPI 34.2–249.3 vs. FDG 1–2.3 and SULavg: FAPi: 33.5–130.8 vs. FDG: 1–2.3) (58). Furthermore, they demonstrated in 2 patients that ^68^Ga-DOTA.SA.FAPi identified additional lesions in the brain that could not be detected on ^18^F-FDG PET/CT, likely due to the lower normal brain parenchyma uptake of ^68^GaDOTA.SA.FAPi. Figure 1 demonstrates a comparison of ^68^Ga-DOTA.SA.FAPi to ^18^F-FDG in the same patient. A recent paper using 68Ga-FAPI-04 in a single patient with lung cancer demonstrated the ability to observe 2 brain metastases unobserved in CT images, thereby changing the patients staging and subsequent management, clearly showing the potential power of its diagnostic ability (65).

### 4.4. Tumor volume delineation and radiation treatment planning

Another useful clinical application for FAPI-PET may be to improve gross tumor volume (GTV) delineation in external-beam radiotherapy (EBRT) planning. ^18^F-FDG and hypoxia imaging ligands have traditionally been the radiotracers of most interest to allow EBRT dose boosts to metabolically active or hypoxic areas (66–70). With selective targeting of FAP, and better imaging characteristics of FAPI-PET, a more accurate delineation of additional areas of interest (i.e., the tumor microenvironment) may be of clinical benefit, such as in head and neck cancer with ^68^Ga-FAPI, where differentiation between residual/recurrent disease and post-chemoradiation fibrosis may be a diagnostic challenge for ^18^F-FDG (71).

Recent work compared automated delineation of seven ^68^Ga-FAPI-04 pancreatic cancer cases against manual segmentation of CECT (72), demonstrating that although a clinical standard, CECT led to high interoperator variability of up to 100% in gross target volume (GTV) definition, with a Dice score coefficient of 0.55–0.65 (amongst 6 oncologists). Improved imaging performance of ^68^Ga-FAPI-04 compared to CECT determined that automated delineation using 2 times the background SUV compared well to manual segmentations on review, and may allow for GTV standardization in certain clinical conditions.

In brain tumors, recent research has described the usefulness and added value of using FAPI-PET ligands in tumor volume delineation in radiation treatment planning in a cohort of 13 glioblastoma patients (50), showing a difference in measured GTV compared to MRI (73). Considering the tumor microenvironment is a significant portion of the tumoral mass, gaining complementary information may prove helpful in delineating the best volumes to be treated with radiation therapy. Of note also is that dose painting and dose escalation are common in radiotherapy regimes involving PET imaging (50), adding a FAPI-specific metabolic GTV may be useful for dose escalation on areas with a strong uptake and also allowing a reduction of dose to healthy tissue by changing the PTV accordingly.

### 4.5. Tumor resection margins

There is a growing utilization of intraoperative MRI to assess for tumor margins and completeness of resection. FAPI-PET radioligands may play an important role in delineating the best tumor resection boundaries preoperatively and then be used intraoperatively with the aid of advanced semi-automated navigation systems. Utilization can also be intraoperative use after tumor resection in order to assess any residual tumor while still in the operating room assisting surgeons similar to a frozen section. This may be helpful especially in difficult cases with tumors near eloquent areas.

### 4.5. Biopsy guidance

FAPI-PET may also prove useful in biopsy planning given the heterogeneous uptake in the tumor and the importance of FAP for invasive growth, in turn suggestive of the presence of active tumor cells in areas with high tracer uptake (50). Choosing the right biopsy site can prove difficult with the current standard of care (MRI). Considering tumor heterogeneity and associated peritumoral changes seen on anatomical imaging the biopsy specimen may not be the most representative of tumor aggressiveness. FAPI-PET has been reported to be associated with tumor invasiveness and hence may be suitable to map the tumor pre-biopsy (74). It may also be interesting to have a complete tumor phenotype with a complementary role of hypoxia, glucose and amino acid metabolism radiotracers when used together. With newer PET scanning technology such as digital PET and large field of view imaging (75, 76), the use of lower radiopharmaceutical doses is possible and radiation exposure would be minimal with the main limiting factors being cost and availability of FAPI-PET radioligands.

### 4.6. Recurrence

Brain tumor recurrence is, at the moment, inevitable in a large number of cases. Differentiating progression from pseudoprogression as well as subtle or early true recurrence vs. post-treatment changes can be challenging with MRI. PET techniques especially with aminoacid imaging offer a proven advantage. FAPI-PET offers an additional tool in the armamentarium of the oncologist or neurosurgeon. Important work is in progress.

### 4.7. Therapy and theranostics

FAP has been touted as an attractive treatment target for oncology in brain tumors and other cancers. Since FAPI is showing significant strides one may envision a role for FAPI theranostics in the treatment of brain tumor patients. While the therapeutic application of FAPI tracers is still limited thus far, some studies have been carried out. A trial in 26 patients came about to make use of high FAP expression as a therapeutic target, for primarily metastatic colorectal cancer using a sibrotuzumab antibody (77). Testing of antibodies developed against FAP and labeled with ^177^Lu was also carried out in preclinical models (78, 79). Promising efficacy results were observed. FAP-targeting immunotherapy agents (80), vaccines (81) and FAP-specific nanoparticles (82) are also under active research.

A recent clinical investigation has investigated theranostic pairing of experimental ^90^Y-FAPI-46 coupled with ^68^Ga-FAPI-04 in a patient with metastasized breast and colorectal cancer with peritoneal metastases. Imaging with ^68^Ga-FAPI-04 revealed high tracer uptake in metastases and a reduction in pain symptoms after therapy with a 3-cycle regime (7.4 GBq per cycle) of experimental ^90^Y-FAPI-46. The patient responded with stable disease related to breast cancer, and remission of peritoneal lesions, before eventually progressing some months later. As well as ^90^Y-FAPI-46 as a therapeutic option, development is also ongoing for ^188^Re FAPI (59). Although still very much in its infancy, this case study illustrates the theranostic potential of FAPI radioligands and it is expected that more efforts will be directed towards brain cancers (83).

## 5. Benefits

In summary, FAPI Brain PET may be helpful in:

• Primary brain tumor assessment/prognostication and brain metastasis detection
• Tumor volume delineation and radiation treatment planning
• Tumor surgical resection margins and completeness
• Biopsy guidance
• Suspicious recurrence assessment
• Therapy and Theranostics

*In vivo* visualization of the tumor microenvironment is made possible by FAPI-PET, resulting in better understanding of tumor heterogeneity and a crucial piece of tumor aggressiveness and invasiveness. FAPI-PET may overcome limitations of other radiotracers including tumors with low FDG-avidity and result in some cases of upstaging through the detection of unknown distant metastases. It may also improve target volume delineation for radiotherapy planning in conjunction with other radiotracers and MRI. FAPI-PET may also complement other PET probes by offering a comprehensive phenotypic assessment of primary brain tumors in adjunct to hypoxia and amino acid metabolism tumor mapping. Multitracer evaluations may be in the future an important piece of brain tumor evaluation and characterization.

## 6. Limitations

As other radioligands, FAPI may not be perfect and have some minimal limitations. Considering FAP is overexpressed mainly in CAFs and not in tumor cells, FAPI-PET may not always reflect the actual tumor microenvironment. A clinical study using ^68^Ga-FAPI-04 and ^68^Ga-FAPI-46 reported non-specific uptake at sites where patients had scarring or degenerative lesions, which lead to false-positive tumor detection (84), although expert reading may alleviate this issue. Similarly, in another study, for one patient with pancreatic cancer who had developed chronic peritumoral inflammation as a result of radiotherapy, the FAPI tracer was unable to accurately identify the target volume for radiotherapy (49).

Additionally, at present only data from single-center studies of small cohorts of patients is available. As a result, in studies where findings are unexpected, larger sample studies are needed to confirm these results. An example of this is a study where sites with low or moderate FAP expression (i.e., the uterus and breast) showed higher FAPI tracer uptake (84). Additionally, ongoing discussions continue on the controversies associated with using FAPI-PET for the diagnosis of bone and lymph node metastases (85–87). Pharmacokinetics with fast clearance from blood and short retention in tumors may be problematic for radionuclide therapy applications or be an advantage allowing closer follow-up cycles with lower toxicity. Hence, structural modification of FAPI optimizing tumor uptake and tumor retention time for peptide radionuclide therapy (PRRT) is another key research direction.

## 7. Conclusions

FAPI-PET is a promising tool for the assessment of primary and metastatic brain tumors and offers several clinical advantages including added sensitivity, management guidance as well a more complete phenotypic evaluation of tumors. Promising research shows the potential for translation of FAPI imaging agents to the development of therapy agents in the realm of theranostic nuclear medicine. Although data is currently sparse, new studies demonstrate FAPI as a viable target for brain PET imaging either as a single probe or within a multi-tracer tumor mapping paradigm, however large prospective trials are needed.
